# Application of machine learning standardized integral area algorithm in measuring the scoliosis

**DOI:** 10.1038/s41598-023-44252-x

**Published:** 2023-11-07

**Authors:** Shuman Han, Hongyu Zhao, Yi Zhang, Chen Yang, Xiaonan Han, Huizhao Wu, Lei Cao, Baohai Yu, Jin-Xu Wen, Tianhao Wu, Bulang Gao, Wenjuan Wu

**Affiliations:** 1 Department of Radiology, The Third Affiliated Hospital of Hebei Medical University, Shijiazhuang 139 Ziqiang Road, Shijiazhuang, 050051 Hebei China; 2 Hebei University of Science and Technology, Shijiazhuang, 050051 Hebei China

**Keywords:** Computational biology and bioinformatics, Diseases, Medical research

## Abstract

This study was to develop a computer vision evaluation method to automatically measure the degree of scoliosis based on the machine learning algorithm. For the X-ray images of 204 patients with idiopathic scoliosis who underwent full-spine radiography, histogram equalization of original image was performed before a flipping method was used to magnify asymmetric elements, search for the global maximum pixel value in each line, and scan local maximal pixel value, with the intersection set of two point sets being regarded as candidate anchor points. All fine anchors were fitted with cubic spline algorithm to obtain the approximate curve of the spine, and the degree of scoliosis was measured by the standardized integral area. All measured data were analyzed. In manual measurement, the Cobb angle was 11.70–25.00 (20.15 ± 3.60), 25.20–44.70 (33.89 ± 5.41), and 45.10–49.40 (46.98 ± 1.25) in the mild, moderate and severe scoliosis group, respectively, whereas the value for the standardized integral area algorithm was 0.072–0.298 (0.185 ± 0.040), 0.100–0.399 (0.245 ± 0.050), and 0.246–0.901 (0.349 ± 0.181) in the mild, moderate and severe scoliosis group, respectively. Correlation analysis between the manual measurement of the Cobb angle and the evaluation of the standardized integral area algorithm demonstrated the Spearman correlation coefficient r = 0.643 (*P* < 0.001). There was a positive correlation between the manual measurement of the Cobb angle and the measurement of the standardized integral area value. Two methods had good consistency in evaluating the degree of scoliosis. ROC curve analysis of the standardized integral area algorithm to measure the degree of scoliosis showed he cutoff value of the standardized integral area algorithm was 0.20 for the moderate scoliosis with an AUC of 0.865, sensitivity 0.907, specificity 0.635, accuracy 0.779, positive prediction value 0.737 and negative prediction value 0.859, and the cutoff value of the standardized integral area algorithm was 0.40 for the severe scoliosis with an AUC of 0.873, sensitivity 0.188, specificity 1.00, accuracy 0.936, positive prediction value 1 and a negative prediction value 0.935. Using the standardized integral area as an independent variable and the Cobb angle as a dependent variable, a linear regression equation was established as Cobb angle = 13.36 + 70.54 × Standardized area, the model has statistical significance. In conclusion, **t**he integrated area algorithm method of machine learning can quickly and efficiently assess the degree of scoliosis and is suitable for screening the degree of scoliosis in a large dataset as a useful supplement to the fine measurement of scoliosis Cobb angle.

## Introduction

In recent years, the incidence of idiopathic scoliosis (AIS) in children and adolescents has gradually increased in some areas, causing serious adverse consequences for the growth and development of patients^1–4^. Further progress of the disease will result in head and neck deflection, unequal height of shoulders, pelvis tilt and rotation, and unequal length of lower limbs, with different degrees of compression on tissues and organs^5–9^. Severe cases of scoliosis need spinal surgery, and the surgery and postoperative complications may cause great pain, infection, mortality, proximal junctional kyphosis, and neurological disorders to the patients^10,11^. Therefore, early detection and correction of scoliosis is the key to its prevention and control. The Cobb angle is the standard evaluation index of spinal curvature angle^12–15^. Richards et al. believed that Cobb angle 25° was the starting angle for brace treatment^16^, and Tan et al. held the review that a Cobb angle of 25° at presentation had the best overall predictive value for curve progression to a Cobb angle of 30° or more at skeletal maturity^17^. Moreover, surgical treatment should be performed for patients with a Cobb angle of 45°–50°^18^. Based on the relevant literature^16–18^ and the guidelines of the Scoliosis Research Society (https://www.srs.org/patients-and-families/conditions-and-treatments/adolescents/treating-scoliosis) for the treatment of different scoliosis, our study divided the severity of scoliosis into four grades based on the Cobb angle: normal when Cobb angle is ≤ 10°, mild when 10° < Cobb angle ≤ 25°, moderate when 25° < Cobb angle ≤ 45°, and severe when Cobb angle is > 45°. Compared with computed tomography (CT) and magnetic resonance imaging (MRI), full spine orthopedic radiography has a low medical cost and simple operation and can obtain more comprehensive and rich diagnostic information in one single examination, which is suitable for large-scale screening of scoliosis. However, the manual measurement of the Cobb angle in large quantities has high time and labor costs, and manual Cobb angle measurement requires experience and judgment^19^, with high inter-observer and intra-observer variability^20^. Use of the computer vision technology can help to extract more diagnostic information than human eye observation. Radiologists have explored the use of artificial intelligence to analyze medical images to improve the accuracy and efficiency of disease diagnosis^21–24^. The principle of measuring the Cobb angle determines that it can be used to effectively evaluate local scoliosis angles, but there are currently no research reports on evaluating scoliosis from a global perspective. Global evaluation, especially for cases with double spinal curves, provides a more comprehensive perspective and helps to better understand and manage scoliosis. If the area of the deviation of the spine curve from the central axis and the degree of curvature from the overall global perspective of the spine curve can be measured, it will be a good supplement to local evaluations such as using the Cobb angle measurement. Moreover, manual measurement of the Cobb angle in a large number of patients is time consuming and needs a lot of work besides experience and correct judgment^19^, with high inter-observer and intra-observer variability^20^. Computer vision technology can extract more diagnostic information than human eye observation, and radiologists are exploring the use of artificial intelligence to analyze medical images to improve the accuracy and efficiency of disease diagnosis. Based on computer digital image processing, this study developed an artificial intelligence machine learning algorithm for searching the maximal pixel and local extreme value of pixels in the row of the whole spine X-ray image and used the standardized integral area value to measure the degree of scoliosis, so as to quickly and accurately realize computer automatic evaluation of the degree of scoliosis.

## Materials and methods

### Subjects

This retrospective one-center study was approved by the ethics committee of the Third Hospital of Hebei Medical University. For this study, informed consent has been waived by the Third Hospital of Hebei Medical University due to the anonymity and retrospective nature of the study. All methods were performed in accordance with the relevant guidelines and regulations. The full-spine X-ray digital images of patients with AIS were collected. The inclusion criteria were no history of spinal surgery and other spinal diseases. The Cobb angle was measured manually by a senior radiologist. The imaging in each patient was measured once every month for a total of three times by a rater with 15-year experience on X-ray diagnosis, and the average of three measurements was taken as the reference standard.

### X-ray digital image processing

The MATLAB 2018B (Matrix Laboratory, version number R2018B) image processing toolbox design software was used to program machine learning algorithms and calculate and operate digital matrices.

Pre-processing of the original X-ray digital image was conducted using four points to circle the approximate position of scoliosis and perform flipping operation and histogram equalization transformation to highlight the area where the spine was located. As shown in formula (1), if the coordinates of the four points recorded were (x_i_, y_i_:) with i = 1, 2, 3, and 4, then the left, right, upper, and lower boundaries of the spine region selection box were:

1 $$ \begin{aligned} & B_{left} = \min \left( {{\text{x}}_{{\text{i}}} } \right),\;\;B_{right} = \max \left( {{\text{x}}_{{\text{i}}} } \right), \\ & B_{up} = \max \left( {{\text{y}}_{{\text{i}}} } \right),\;\;B_{low} = \min \left( {{\text{y}}_{{\text{i}}} } \right),\quad {\text{i}} = 1,2,3,4 \\ \end{aligned} $$

The pixel matrix of the selected spine region was marked as W.

(2) Flip overlay algorithm formula: This was to prevent confusion of high and low pixels of the original pixel points after overlay. Each pixel of the pixel matrix W of the spine region selection box image was set to halve and change to matrix X:(x_i,j_), i = 1, …, M; J = 1, …, N as shown in formula (2):

2 $$ x\_translation = i - \left[ \frac{M}{2} \right],\;\;y\_translation = j - \left[ \frac{N}{2} \right], $$

The superimposed matrix G was shown in formula (3):

3 $$ G(i,j) = x_{i,j} + x_{i - 2 \times x\_translation,j - 2 \times y\_translation} $$

**Figure 1:**
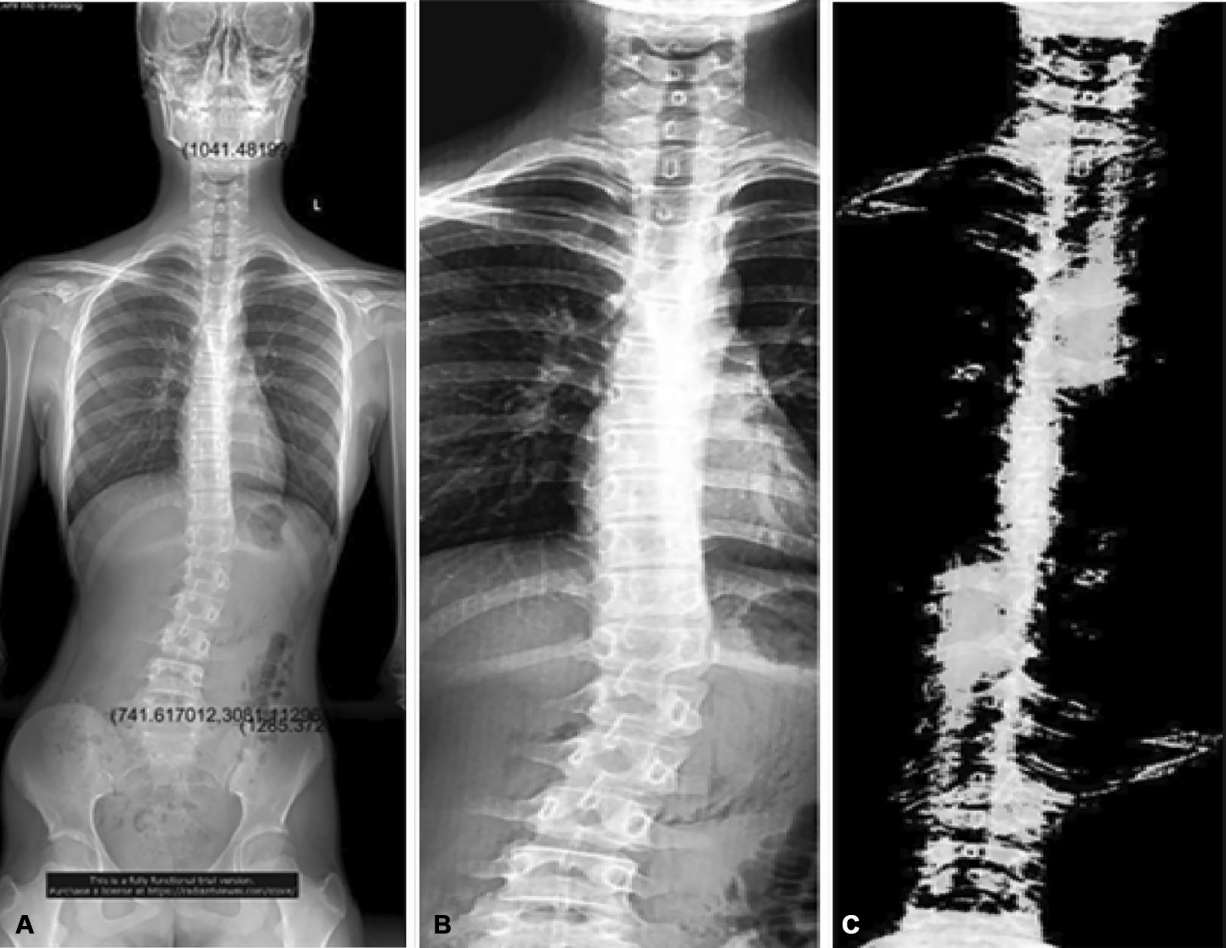
Spinal radiograph of the full spine, enhanced spine, and symmetrical superposition of the spinal region. (**A**) Full spine upstanding radiograph. (**B**) Enhancement image of the spinal region. (**C**) Spinal symmetric superposition imaging.

In the flipped superimposed image, when the pixel cutoff was equal to 0.5, the pixel higher than the cutoff was converted to 1, and the pixel lower than the cutoff was converted to 0. The obtained binary image could effectively remove the imaging pixel with central symmetry, while retaining the asymmetric and deformed side-bending image as shown in Fig. 1.

**Figure 2:**
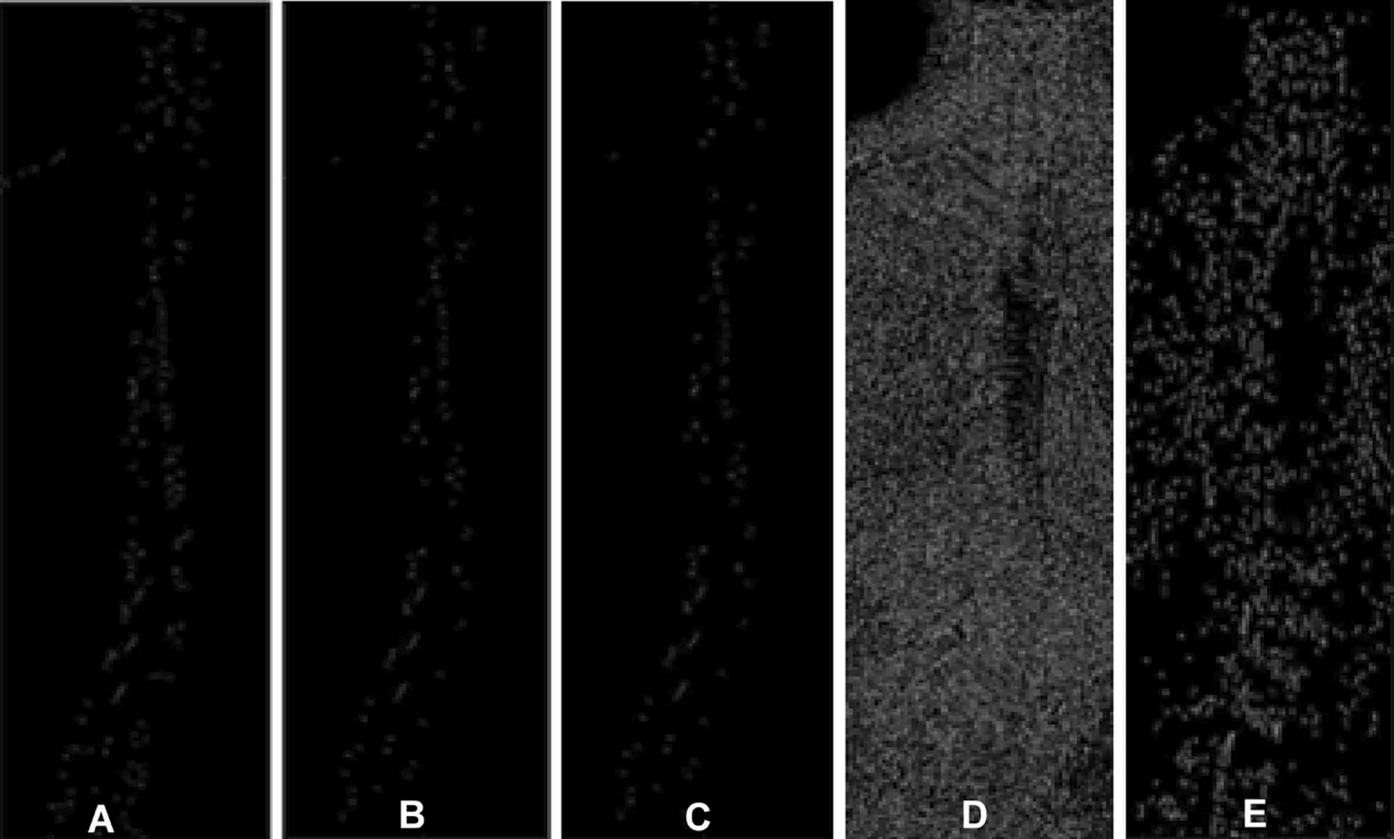
Extraction of global and local maximal pixels in each row. (**A**) Pixel maximal point plot per row. (**B**) The maximal point is opened in each line. (**C**) Corrosion diagram. (**D**) Graph of local pixel extreme points. (**E**) Open operation diagram of local pixel extreme points.

Extracting the global maximal pixel and local maximal pixel in each line: The pixel with the maximal brightness in each row was searched as the global maximal pixel, and the local extreme point was searches with the pixel in each row higher than 3–5 pixels on both sides to extract the maximal pixel in each row so as to perform the open operation and corrosion operation. In the opening operation, objects with an area less than 3 in the binary image were deleted. According to the nature of the X-ray image of the whole spine, the square area was used for noise removal during the corrosion operation as shown in Fig. 2.

Selection of candidate anchor points and fine anchor points of scoliosis line by the sieve method.

**Figure 3:**
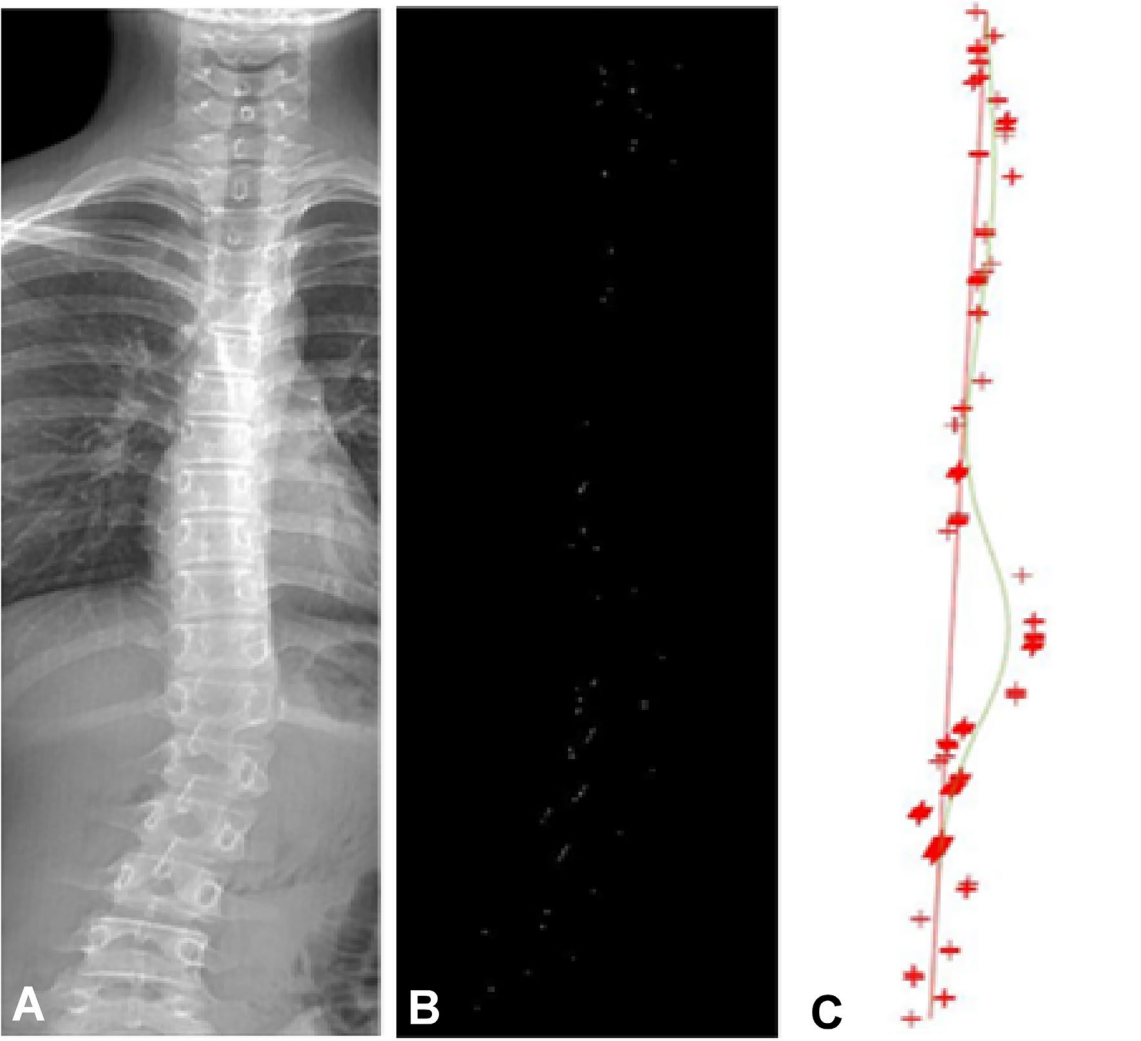
Cubic spline smooth scoliosis diagram at fine anchor points. (**A**) Enhancement of the spinal area. (**B**) Candidate anchor points obtained from intersection. (**C**) Fine anchor points and cubic spline smooth scoliosis plot.

After the histogram was balanced, the symmetric overlay threshold algorithm was used to highlight the asymmetric pixels, search for the pixel point with the maximal brightness in each row, and then find the local extreme point where the pixel in each row was higher than 3–5 pixels on both sides. The intersection of the two points served as a candidate anchor. Start from the top candidate anchor and connect a straight line to the candidate anchor in the second row. If the absolute value of the line slope was greater than 1, the point in the second row would be used as the fine anchor, otherwise continue to search for the candidate anchor in the next row. All fine anchor points were fitted with the cubic spline algorithm, and the approximate curve of the spine was finally obtained. The standardized area between this curve and the straight line connecting the head and tail of the spine was calculated, and the degree of scoliosis with the standardized area was measured (Fig. 3).

Area integral standardization: The first and last points of the above side curve were connected to get the reference line, and 100 equally spaced parallel lines were drawn horizontally. The length of the parallel line segments between the side curve and the reference line was added to get the estimated value of the area between the side curve and the reference line. The horizontal distance between the leftmost point and the rightmost point of the side curve in the horizontal direction were used as the standardization factor, and the above area was divided by the standardization factor to obtain the standardized area, as shown in formula (4):

4 $$ normlizedarea = \frac{{\sum\nolimits_{i = 1}^{100} {len_{i} } }}{\max (x) - \min (x)} $$

where X is the independent variable value range of the side bending curve y = f(x), and len_i_ was the length of the ith parallel segment between the side bending curve and the reference line.

### Statistical analysis

The SPSS22.0 statistical software was used to analyze the data, and the Spearman correlation analysis was used to evaluate the correlation between the manually measured Cobb angle and the standardized integral area algorithm. According to the standard of manual measurement of the Cobb angle, the test cases were divided into three groups of mild (10° < Cobb angle ≤ 25°), moderate (25° < Cobb angle ≤ 45°), and severe (Cobb angle is > 45°) scoliosis, and the area under curve (AUC), sensitivity and specificity of the receiver operating characteristic curve (ROC) curve for evaluating the degree of scoliosis were analyzed using the ROC curve. The reference value for evaluation of the standardized integral area algorithm was determined by referring to the Youden index, the cutoff value with the highest sensitivity was selected for judging the moderate scoliosis, and the cutoff value with the highest specificity was selected for judging the severe scoliosis. The linear regression analysis was conducted using the standardized integral area as an independent variable and the Cobb angle as a dependent variable. *P* < 0.05 was set as statistically significant.

## Results

Totally, 204 patients were enrolled (Table 1), including male 45 and female 159 aged 15.65 ± 6.01 (7–38) years. There were thoracic main curvature in 64 (31%) patients, lumbar main curvature in 60 (29%), thoracolumbar main curvature in 30 (15%), and double main curvature in 50 (25%). Treatment was performed with brace in 87 (43%) patients and surgery in 19 (9%) besides observation in 98 (48%). In the grading of Risser sign, there were sign I in 15 (7%) patients, sign II in 21 (10%), sign III in 39 (19%), sign IV in 54 (27%), and sign V in 75 (37%).

**Table 1:** Baseline data of patients with scoliosis.

Variables	Data
Patients (n, mean ± standard deviation)	
No. of cases	204
M/F	45/159
Age (y)	7–38 (15.65 ± 6.01)
Scoliosis features (n, %)	
Thoracic main curvature (n, %)	64 (31%)
Lumbar main curvature (n, %)	60 (29%)
Thoracolumbar main curvature (n, %)	30 (15%)
Double main curvature (n, %)	50 (25%)
Treatment (n, %)	
Observation (n)	98 (48%)
Brace (n, %)	87 (43%)
Surgery (n, %)	19 (9%)
Risser sign (n, %)	
Risser sign I (n, %)	15 (7%)
Risser sign II (n, %)	21 (10%)
Risser sign III (n, %)	39 (19%)
Risser sign IV (n, %)	54 (27%)
Risser sign V (n, %)	75 (37%)

The software designed by the MATLAB 2018B image processing toolbox in this study needs 4 GB of running memory, and about 10 s was needed to processes an image and calculates the standardized area. Processing an image and calculating the standardized integral area took about 6.5 ± 0.2 s.

**Figure 4:**
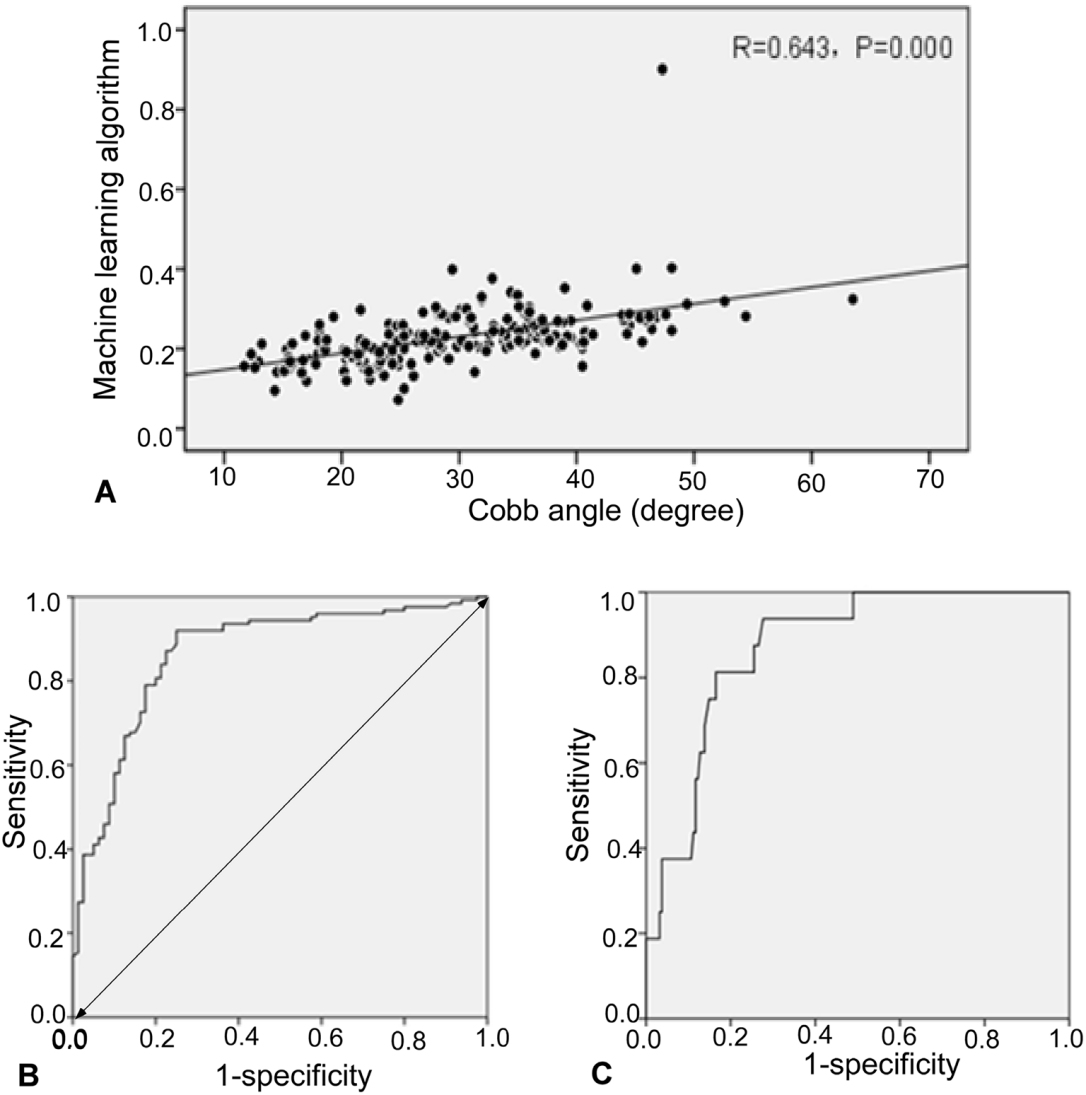
Spearman correlation analysis and receiver operating characteristics (ROC) curve analysis. (**A**) Spearman correlation analysis diagram of manual Cobb Angle measurement and standardized integrated area algorithm of machine learning. (**B**) ROC curve analysis of standardized integral area algorithm for measuring the degree of moderate scoliosis. (**C**) ROC curve analysis of standardized integral area algorithm to measure the degree of severe scoliosis.

In manual measurement, the Cobb angle was 11.70–25.00 (20.15 ± 3.60), 25.20–44.70 (33.89 ± 5.41), and 45.10–49.40 (46.98 ± 1.25) in the mild, moderate and severe scoliosis group, respectively, whereas the value for the standardized integral area algorithm was 0.072–0.298 (0.185 ± 0.040), 0.100–0.399 (0.245 ± 0.050), and 0.246–0.901 (0.349 ± 0.181) in the mild, moderate and severe scoliosis group, respectively (Table 2). Correlation analysis between the manual measurement of the Cobb angle and the evaluation of the standardized integral area algorithm (Table 2 and Fig. 4) demonstrated the Spearman correlation coefficient r = 0.643 (*P* < 0.001). There was a positive correlation between the manual measurement of the Cobb angle and the measurement of the standardized integral area value. Two methods had good consistency in evaluating the degree of scoliosis.

**Table 2:** Correlation analysis of manually-measured Cobb angle with standardized integral area algorithm.

Groups	No.	Standardized integral area value (range/mean)	Cobb angle (range, mean, degree)
Mild scoliosis	80	0.072–0.298 (0.185 ± 0.040)	11.70–25.00 (20.15 ± 3.60)
Moderate scoliosis	108	0.100–0.399 (0.245 ± 0.050)	25.20–44.70 (33.89 ± 5.41)
Severe scoliosis	16	0.246–0.901 (0.349 ± 0.181)	45.10–49.40 (46.98 ± 1.25)
Total	204	0.072–0.901 (0.237 ± 0.08)	12.92–49.40 (32.02 ± 8.57)
*r* value	0.643		
*P*	0.000

ROC curve analysis of the standardized integral area algorithm to measure the degree of scoliosis is shown in Table 3, Fig. 4B and C. The cutoff value of the standardized integral area algorithm was 0.20 for the moderate scoliosis with an AUC of 0.865, sensitivity 0.907, specificity 0.635, accuracy 0.779, positive prediction value 0.737 and negative prediction value 0.859, and the cutoff value of the standardized integral area algorithm was 0.40 for the severe scoliosis with an AUC of 0.873, sensitivity 0.188, specificity 1.00, accuracy 0.936, positive prediction value 1 and a negative prediction value 0.935. Using the standardized integral area as an independent variable and the Cobb angle as a dependent variable, a linear regression equation was established as Cobb angle = 13.36 + 70.54 × Standardized area, the model has statistical significance.

**Table 3:** ROC analysis of standardized integral area algorithm for measuring scoliosis.

Variables	Standardized integral area cutoff value	AUC	Sensitivity	Specificity	Accuracy	PPV	NPV
Moderate scoliosis	0.20	0.865*	0.907	0.635	0.779	0.737	0.859
Severe scoliosis	0.40	0.873*	0.188	1.00	0.936	1.00	0.935

Significant values are in underline.

*ROC* receiver operating characteristics, *AUC* area under the ROC curve, *PPV* positive predictive value, *NPV* negative predictive value.

*P < 0.05.

## Discussion

Our study found that the standardized integral area algorithm acted well in measuring the mild, moderate and severe scoliosis with good consistency with the manual measurement of the Cobb angle. ROC curve analysis revealed high diagnostic values of the standardized integral area algorithm in evaluating the moderate and severe scoliosis.

The purpose in evaluation of the scoliosis degree is to guide selection of clinical treatment plans. At present, the evaluation methods of the scoliosis degree mainly include imaging Cobb angle measurement method, physical examination method, and image detection method^25–28^. Physical examination methods include torso rotation angle method, Adams forward flexion test, and spine measurement instrument method^26^. Image detection methods mainly include Moire local measurement method and laser scanner method^27,28^. These methods have poor measurement accuracy, complex operation steps, and cannot obtain comprehensive diagnostic information. For judging the severity of scoliosis, the clinical standard is the maximal Cobb angle value measured on the X-ray film of the whole spine. During large-scale census, manual measurement of the Cobb angle is tedious and time-consuming, which is easy to cause a certain degree of subjective error^29^, affecting the classification of scoliosis and the choice of treatment strategy. In order to avoid errors caused by subjective factors of doctors and improve work efficiency, computer image processing technology is used to automatically, quickly and accurately evaluate the degree of scoliosis, which has an important significance for large-scale screening of adolescent scoliosis.

Among the 204 patients enrolled in this study, the standardized integral area value was ≤ 0.2, 0.2–0.4, and ≥ 0.4 for the mild, moderate and severe scoliosis, respectively. Taking the manual measurement method of the Cobb angle as the standard, the AUC of the integral area calculation method based on the machine learning was 0.865 and 0.873 for the moderate and severe scoliosis, respectively, which has achieved high diagnostic efficiency among similar studies^30,31^. The core algorithm in this study is the integral area algorithm of the maximal pixel in the row and the local extreme value of the pixel. By scanning the digital image line by line, the maximal pixel point was found as the global maximal pixel point, and the pixel point in each row that was higher than 3–5 pixels on both sides was found as the local maximal pixel point. The left and right pixels of the pixel point were symmetrically distributed, and the intersection of the two points was used as the candidate anchor point. The sieve method starts from the first candidate anchor point of the cervical spine to connect the candidate anchor points in the lower row with a straight line. If the slope is less than 1, it will be discarded, because the adjacent row of spine cannot be deformed to a nearly horizontal degree with a slope of less than 1. The candidate anchor points in the following row are screened out, and the candidate anchor points in the next line continue to be connected until a reasonable candidate anchor point is screened and listed as a fine anchor point. In this study, the true anchor points were screened by setting the standard screen method with a line slope of 45°, and then the cubic spline algorithm was used to fit all the fine anchor points to obtain the cubic spline smooth spine line graph, which realized the true reproduction of the curved spine curve. The algorithm takes the horizontal distance between the leftmost point and the rightmost point of the scoliosis curve in the horizontal direction as the standardization factor, divides by the standardization factor to obtain the standardized area, and evaluates the degree of scoliosis by measuring the area deviating from the central axis. The algorithm in this study converts the manual measurement process of Cobb angle into the machine learning calculation process of feature pixel area, which improves the detection speed and reduces the repeated labor intensity of the measuring doctor. The reason why the central sacral line (CSL) was not used as the midline in the study was that the CSL is an indicator for evaluating the degree of coronal balance on images. Using CSL as a reference line to obtain another standardized area to evaluate coronary imbalance is a promising idea. However, the purpose of our study was to evaluate the degree of scoliosis from a global perspective, which is an “angle” evaluation in another sense, rather than evaluating coronal balance.

The method innovation of this study lies in the selection of fine tracing points. Through multiple fitting verification, it is found that the leftmost anchor point in the left, middle and right positions has the best fitting effect. The reason is that the bending curve is located on the left side of the centerline, that is, the overall offset area is to the left, resulting in the best fitting effect of the anchor point on the left. The measurement of the maximal Cobb angle commonly used in clinic only describes the extreme value of the degree of scoliosis, and the Cobb angle formed by the rest of scoliotic vertebral body also has important diagnostic value but with a large measurement workload. Different from measurement of the maximal Cobb angle, for patients with double or multiple spinal curves, the cubic spline smooth line graph of the integral area method intuitively displays the position deviation and the degree of deviation of the abnormal curvature, highlights the position information of scoliosis, and intuitively displays the degree of curvature, which is conducive to the evaluation of scoliosis from a global perspective. The orientation and size of the abnormal area of the deviation from the central line can be used as an important reference for the selection of treatment plans and the evaluation of treatment effects. The algorithm in this study ensures the accurate positioning of the spine landmarks, proves the biomechanical changes of the scoliosis spine with mathematical methods, and realizes the unification of the mathematical and clinical significance of the algorithm.

In similar studies, Glocker et al. automatically located and identified the spine on CT images based on regression forest and probability map models, with a recognition rate of 81%^32^. Their subsequent studies improved robustness by using supervised classification of forests, but still used semi-automatic markers, which could not achieve real-time evaluation^33^. Hao et al. based on the random forest algorithm of 3D CT image, used regression and comprehensive scoring to locate the spine center and then used the CNN depth learning model for further correction^34^. The 3D image provides rich position information and texture information to obtain high recognition accuracy, but the CT is a lying non-weight bearing image, and the spine is not subject to gravity and pressure, thus greatly reducing the degree of scoliosis. The result does not conform to clinical practice and cannot guide treatment. Thalengala et al.^35^ proposed a machine learning method to perform threshold and binary logic operations after image fast inversion transformation. Gaussian filtering was used in their study to obtain the boundary of the spine, which can easily and quickly extract the contour of the spine. However, the fast Fourier transform interpolation used by Thalengala et al.^35^ has a large error under non-integral period sampling. The use of cubic spline interpolation in this study can effectively reduce the impact of non-integral period sampling on Fourier transform, with high detection accuracy being obtained. In the past, these computer machine learning algorithms have some problems, such as low detection accuracy and high computational overhead, which cannot meet the needs of large-scale screening. The algorithm of our study improved these defects and realized the computer vision scoliosis degree evaluation with high efficiency and low computational cost.

In our study, some inconsistent results of scoliosis were demonstrated in the two methods of evaluation: when the degree of scoliosis was mild in manual evaluation of the Cobb angle, its integral area was in the moderate range of 0.2–0.4; when the degree of scoliosis evaluated by measuring the Cobb angle was moderate, its integral area was within the mild range of less than 0.2. The linear regression analysis has enabled us to recognize the synergy and differences between the Cobb angle evaluation and the standardized integral area value evaluation, with the most important being a deeper understanding of the differences. It is precisely this difference that has led to the occurrence of inconsistent results in evaluating scoliosis between the two methods. The reason is that the marginal value of Cobb angle grading has an impact on the evaluation results of the integral area algorithm. The Cobb angle measured in cases with inconsistent results of the two methods is just the critical value for measuring the mild and moderate grades of scoliosis. In our study, the Cobb angle was used as the standard to evaluate the measurement accuracy of the machine learning algorithm, but the Cobb angle evaluation method was based on measuring the included angle of scoliotic vertebral body, focusing on displaying the local characteristics of scoliotic vertebral body. The machine learning algorithm was used to calculate the area of the deviation of the spine curve from the central axis, and the degree of curvature was measured from the overall mean of the spine curve. The machine learning algorithm can be used as a good supplement to local evaluations using the Cobb angle measurement. Moreover, this machine learning method can be embedded in the reporting system as an artificial intelligence method for evaluating the degree of scoliosis, which plays an important role in improving work efficiency in the clinical practice. The two methods in this study have the same judgment results for most cases, and each has its own advantages and disadvantages. In the screening work, the two methods can be organically combined, and the work strategy of computer large-scale screening and manual key measurement can be adopted.

In our study, the sensitivity of the standardized integral area algorithm in predicting severe scoliosis was decreased. The anchor points obtained by the algorithm actually included some noise points, some of which deviated significantly to the left or right. If such points were allowed to exist, the cubic spline smooth scoliosis plot of mild to moderate scoliosis patients would be biased by certain noise points, resulting in a large standardized area and loss of accuracy. Based on the experience of selecting fine anchors for most samples, the angle between the connecting line of continuous fine anchors and the y-axis would not exceed 45°. Once the degree was greater than 45°, we would abandon this anchor and instead searched for a lower anchor to connect. If this anchor met the requirements, it would be selected as a fine anchor and truly participated in the connection. We had attempted to use the threshold up to 50° and 60°, both resulting in lower accuracy for mild and moderate angle measurements. So, we adopted the threshold of 45°, which resulted in abandon of the continuous anchor lines which were truly greater than 45° because of exceeding the threshold. Thus, some smaller angle points were found as fine anchor points, resulting in underestimation of the standardized integral area. Severe scoliosis is very eye-catching and can be easily distinguished by human vision. Correct evaluation of mild to moderate scoliosis is related to whether specific treatment measures can be implemented correctly. Different treatment measures such as corrective gymnastics and braces determine the treatment effect, which is the psychological impact on patients. The results of this study showed that the integral area method had a good overall evaluation effect on the degree of mild to moderate lateral bending.

Some limitations existed in this study, including the small sample size, retrospective study design, one-center study, and Chinese patients enrolled only. Considering that the characteristics of severe scoliosis are obviously easy to identify, the proportion of patients with severe scoliosis in the enrolled cases is low. All these issues may produce some bias. Future randomized, prospective, multi-center studies with multiple races of patients enrolled may be able to solve all these issues and obtain better outcomes.

To sum up, this study was based on machine learning to search the maximal pixel and local extreme value of pixels in the row of the whole spine X-ray image, screen the fine anchor points with the sieve method, extract the important features of the curvature of the spine and carry out curve recognition, thus achieving accurate recognition and positioning of the spine by calculating the standardized area of the deviation of the curvature of the abnormal bending spine from the axis and the degree of scoliosis by the size of the area. This algorithm has the advantages of fast detection speed, high accuracy and low computational cost, greatly improving the efficiency of batch processing of full spine X-ray images, and making a simple and rapid assessment of scoliosis severity. It can be used as an important supplement to the Cobb angle evaluation of scoliosis, reduce the complicated work of the measuring physician, avoid fatigue errors, and avoid the measurement errors of artificial evaluation.

## Data Availability

The datasets generated and/or analysed during the current study are not publicly available due to the fact that these data are owned by the hospital but are available from the corresponding author on reasonable request.
